# Risk factors of bleeding in patients with atrial fibrillation undergoing percutaneous coronary intervention: an analysis from the MANJUSRI study

**DOI:** 10.3389/fcvm.2026.1752209

**Published:** 2026-05-18

**Authors:** Yang Li, Wenbin Lu, Yu Wang, Lijuan Chen, Genshan Ma

**Affiliations:** Department of Cardiology, Zhongda Hospital, School of Medicine, Southeast University, Nanjing, China

**Keywords:** anticoagulant, atrial fibrillation, bleeding, risk factors, ticagrelor

## Abstract

**Background:**

The risk factors of bleeding related to dual or triple therapy in patients with atrial fibrillation after percutaneous coronary intervention (PCI) are not fully explored. We addressed this issue on patients enrolled in the MANJUSRI trial.

**Methods:**

Data of patients assigned to the dual therapy group (ticagrelor + warfarin, Dual group) and the triple group (clopidogrel + aspirin + warfarin, Triple group) in the MANJUSRI trial were analyzed. Multivariate regression analysis was used to determine the risk factors of bleeding among enrolled patients.

**Results:**

The incidence of overall bleeding at 6 months was 36.49% in the dual group and 35.62% in the triple group (*P* > 0.05). Multivariate cox regression analysis showed that alcohol consumption was positively correlated with overall bleeding in the total cohort (OR = 3.905, *P* = 0.001) and in the dual therapy group (OR = 4.643, *P* = 0.034). Age was positively associated with overall bleeding risk (OR = 1.059, *P* = 0.032), while body mass index was negatively associated with overall bleeding risk (OR = 0.911, *P* = 0.048) in the triple therapy group.

**Conclusions:**

Alcohol consumption increased the risk of overall bleeding after PCI in atrial fibrillation patients, especially in patients treated with warfarin and ticagrelor. Aging also increased bleeding risk in the whole cohort. Lower body weight contributed significantly to increased risk of overall bleeding in AF patients post PCI receiving combined clopidogrel, aspirin and warfarin medications. Above factors should be considered in terms of individualized anticoagulation management in AF patients post PCI aiming to reduce their bleeding risk.

**Clinical Trial Registration**

https://clinicaltrials.gov/study/NCT02206815, identifier NCT02206815.

## Introduction

Risk of systemic thrombotic events are higher in patients with atrial fibrillation and acute coronary disease (ACS). However, the best choice of antithrombotic strategies for these patients is still under exploration, the balance between the therapeutic effects of preventing thrombotic events and safety profile of avoiding bleeding is the key issue of successful therapy (1–6).

The current guidelines recommend dual antithrombotic treatment with an oral anticoagulant plus a P2Y_12_ inhibitor after discharge for these patients (1, 2). Clopidogrel is the preferred P2Y_12_ inhibitor of the dual therapy. Evidence is accumulating now on the efficacy of ticagrelor as the choice of P2Y_12_ inhibitor in the dual therapy option. MANJUSRI trial was designed to determine the efficacy and safety of ticagrelor in AF patients after PCI (7). The study found that the incidence of total bleeding events in AF patients after PCI treated with ticagrelor was comparable to that in patients treated with clopidogrel plus aspirin, the incidence of cardiovascular events were also comparable between the groups (8). Present analysis aimed to explore the risk factors of bleeding risk in all patients enrolled in the MANJUSRI trial, and in patients treated with ticagrelor therapy plus warfarin group (Dual group) or the clopidogrel plus aspirin and warfarin therapy group (Triple group).

## Materials and methods

### Patients

Patients enrolled in the MANJUSRI trial were randomly assigned to the ticagrelor plus warfarin therapy group (Dual group) or the clopidogrel plus aspirin and warfarin therapy group (Triple group). All patients in the trial are required to be treated with warfarin for at least one year. In the triple therapy group, patients will receive aspirin 100 mg/d and clopidogrel 75 mg/d for at least 6 months after the drug eluting stent implantation. In the warfarin and ticagrelor group, patients will receive ticagrelor 90 mg/bid for at least 6 months after the drug eluting stent implantation. Other cardiac medications will be given at the discretion of the attending physicians. We recommend using PPI in each patient to reduce the gastrointestinal bleeding risk. The primary endpoint is the overall bleeding up to 6 months, according to TIMI criteria and classifications. The secondary endpoints are the major bleeding events up to 6 months, according to TIMI criteria (7). This clinical trial was registered with ClinicalTrials.gov (NCT02206815). Baseline characteristics of patients and bleeding events were described in the main results of the trial (4). The study was approved by the ethics committee of Zhongda Hospital, Southeast University (2014ZDSYLL077.3). All patients provided written informed consent (2015/06/01-2018/12/31).

### Risk factors

Risk factors including sex, age, body mass index, hypertension, diabetes, glomerular filtration rate, current smoking, alcohol consumption, heart failure (ACC/AHA stage C and D), history of stroke, and history of myocardial infarction were analyzed. Definitions for risk factors are based on current international guidelines.

### Statistical analysis

SPSS software (Version 26, IBM) was used for statistics. Cox regression and logistic regression analysis was performed to identify the relationship between risk factors and bleeding risk. *P* < 0.05 was considered statistically significant.

## Results

A total of 294 patients were enrolled in the MANJUSRI trial and 148 patients in the dual therapy group and 146 in the triple therapy group. The average CHA2DS2-VASc score was 3.41 in the ticagrelor therapy group and 3.22 in the Triple therapy group. The mean HAS-BLED scores were 2.01 in the ticagrelor therapy group and 1.97 in the triple therapy group. The mean INR at randomization was 2.08 in the ticagrelor therapy group and 2.13 in the Triple therapy group, respectively. Baseline procedural characteristics were described in detail in our previously published report (8). The incidence of overall bleeding at 6 months was 36.49% in the dual group and 35.62% in the triple group (*P* > 0.05) (8). So the bleeding event was quite the same between the two groups, we thus grouped the patients as bleeding group versus non-bleeding group in this analysis. Baseline characteristics of the study cohort are shown in Table 1. Body mass index in the overall bleeding group was significantly lower than those in the non-overall bleeding group (25.2 vs. 26.5, *P* = 0.015). The proportion of diabetes patients in the overall bleeding group was significantly lower than that in the non-overall bleeding group (21.7% vs. 36.7%, *P* = 0.008).

**Table 1:** Baseline clinical features of overall bleeding group versus non-overall bleeding group.

Baseline characteristics	Overall bleeding group (*n* = 106)	Non-overall bleeding group (*n* = 188)	*P* Values
Female	45 (42.5%)	73 (38.8%)	0.543
Age (years)	70.4	68.6	0.068
BMI (kg/m^2^)	25.2	26.5	0.015
Hypertension	77 (72.6%)	141 (75.0)	0.657
Diabetes mellitus	23 (21.7%)	69 (36.7%)	0.008
Heart failure	34 (32.1%)	80 (42.6%)	0.077
GFR	75.3	71.4	0.088
Smoking	18 (17.0%)	46 (24.5%)	0.135
Alcohol consumption	10 (9.4%)	8 (4.3%)	0.075
Stroke	23 (21.7%)	54 (28.7)	0.188

BMI, body mass index; GFR, glomerular filtration rate (mL/min/1.73 m^2^).

Cox regression analysis showed that alcohol consumption was positively correlated with overall bleeding (*P* = 0.001, OR = 3.905) (Table 2).

**Table 2:** Multivariate cox regression analysis with various models associated with overall bleeding risk.

Covariates	Differences	S.E.	*P* Values	Exp(B)	95%CI Lower	95%CI Upper
Sex	0.025	0.221	0.911	1.025	0.664	1.582
Age (years)	0.383	0.258	0.138	1.466	0.884	2.433
BMI	−0.107	0.236	0.651	0.899	0.566	1.427
Hypertension	−0.193	0.236	0.414	0.825	0.520	1.309
Diabetes mellitus	−0.447	0.254	0.079	0.639	0.388	1.053
Heart failure	−0.137	0.219	0.532	0.872	0.567	1.340
GFR	−0.837	0.476	0.079	0.433	0.170	1.100
Smoking	−0.761	0.309	0.014	0.467	0.255	.857
Alcohol consumption	1.362	0.404	0.001	3.905	1.768	8.626
Stroke	−0.132	0.258	0.608	0.876	0.529	1.452

BMI, body mass index; GFR, glomerular filtration rate (mL/min/1.73 m^2^). Exp (B) is an odds ratio used to predict the probability of bleeding based on a one-unit change in the predictor variable when all other predictors are kept constant.

Subgroup analysis showed that alcohol consumption was also positively associated with overall bleeding risk (*P* = 0.034, OR = 4.643) in the dual therapy group (Table 3).

**Table 3:** Multivariate logistic regression analysis of risk factors associated with overall bleeding risk in the dual therapy group.

Covariates	Differences	S.E.	*P* Values	Exp(B)	95%CI Lower	95%CI Upper
Sex	−0.008	0.424	0.985	0.992	0.432	2.277
Age (years)	−0.002	0.028	0.952	0.998	0.946	1.054
BMI	−0.026	0.060	0.661	0.974	0.866	1.095
Hypertension	0.432	0.503	0.390	1.541	0.575	4.130
Diabetes mellitus	−0.431	0.491	0.381	0.650	0.248	1.702
Heart failure	−0.081	0.420	0.848	0.923	0.405	2.100
GFR	0.011	0.011	0.302	1.011	0.990	1.033
Smoking	−1.283	0.528	0.015	0.277	0.099	0.780
Alcohol consumption	1.535	0.724	0.034	4.643	1.122	19.203
Stroke	−0.363	0.465	0.435	0.695	0.279	1.731

BMI, body mass index; GFR, glomerular filtration rate (mL/min/1.73 m^2^); Exp (B) is an odds ratio used to predict the probability of bleeding based on a one-unit change in the predictor variable when all other predictors are kept constant.

Age was positively associated with overall bleeding risk (*P* = 0.032, OR = 1.059), while body mass index was negatively associated with overall bleeding risk in the triple therapy group (*P* = 0.048, OR = 0.911) (Table 4).

**Table 4:** Multivariate logistic regression analysis of risk factors associated with overall bleeding risk in the triple therapy group.

Covariates	Differences	S.E.	*P* Values	Exp(B)	95%CI Lower	95%CI Upper
Sex	0.476	0.446	0.286	1.609	0.671	3.857
Age (years)	0.057	0.027	0.032	1.059	1.005	1.116
BMI	−0.093	0.047	0.048	0.911	0.831	.999
Hypertension	−0.081	0.472	0.865	0.923	0.366	2.329
Diabetes mellitus	−0.693	0.468	0.139	0.500	0.200	1.252
Heart failure	−0.158	0.423	0.710	0.854	0.373	1.958
GFR	0.014	0.012	0.247	1.014	0.991	1.037
Smoking	0.407	0.626	0.516	1.502	0.441	5.122
Alcohol consumption	0.208	1.363	0.879	1.231	0.085	17.781
Stroke	−0.227	0.464	0.624	0.797	0.321	1.978

BMI, body mass index; GFR, glomerular filtration rate (mL/min/1.73 m^2^); Exp (B) is an odds ratio used to predict the probability of bleeding based on a one-unit change in the predictor variable when all other predictors are kept constant.

## Discussion

Our results showed that alcohol consumption increases the risk of overall bleeding in patients with atrial fibrillation after PCI in the total cohort, especially in the dual therapy group. We have no clear explanation for this phenomenon. Based on this finding, AF patients post PCI should be recommended to restrain the alcohol consumption, especially those receiving dual therapy. Age was positively while body mass index was negatively associated with overall bleeding risk in patients with atrial fibrillation after PCI in the triple group. Our research thus provides a reference for the risk stratification of bleeding among patients with atrial fibrillation after PCI treated with dual or triple strategies. Attention should thus be made on aged and low body weight patients receiving triple therapy.

Current guidelines recommend clopidogrel as the first choice of P2Y_12_ inhibitor as part of dual or triple antithrombotic therapy in patients with atrial fibrillation undergoing PCI, ticagrelor or prasugrel are not recommended due to the lack of clinical evidence (1, 2, 9, 10). However, ticagrelor may be an alternative option for patients with patients with atrial fibrillation undergoing PCI, who also have high thrombotic burden and acceptable bleeding risk (11). Studies demonstrated that the antiplatelet effects of ticagrelor and prasugrel was stronger than clopidogrel, thus might be more effective to prevent thrombotic events (12–14). Few randomized clinical trials compared the efficacy and safety of clopidogrel, ticagrelor and prasugrel in AF patients with acute coronary syndrome or PCI as a part of antithrombotic therapy (15–18). It is suggested that novel P2Y_12_ inhibitor (ticagrelor and prasugrel), as part of dual or triple antithrombotic therapy in patients receiving oral anticoagulants, may be considerable in patients with high risk of ischemia/thrombosis risk (3).

At present, there is no clear clinical evidence on how to screen patients requiring oral anticoagulants who are prone to bleeding post use of ticagrelor. Our study provided clinical data and initial insights in the bleeding risk of patients with atrial fibrillation post PCI and treated with ticagrelor and warfarin, the core finding was that alcohol consumption should be reduced to minimum in these patients to reduce the risk of bleeding, while bleeding monitoring post PCI should be intensified in patients treated with clopidogrel plus aspirin and warfarin in aged and low BMI patients. Future studies are warranted to see if aged and low BMI patients with AF might have less bleeding risk when treated with ticagrelor based medication in comparison with clopidogrel based medication post PCI.

### Limitations

The sample size of this analysis was relatively small. Future studies with larger patient cohort are needed to validate present results.

## Conclusion

Alcohol consumption increased the risk of overall bleeding in atrial fibrillation patients after PCI, especially those treated with ticagrelor. Intensive bleeding monitoring should be applied to aged and low body weight AF patients post PCI treated with triple therapy.

## Data Availability

The original contributions presented in the study are included in the article/Supplementary Material, further inquiries can be directed to the corresponding author.
